# Jumping to the wrong conclusions? An investigation of the mechanisms of reasoning errors in delusions

**DOI:** 10.1016/j.psychres.2014.05.051

**Published:** 2014-10-30

**Authors:** Suzanne Jolley, Claire Thompson, James Hurley, Evelina Medin, Lucy Butler, Paul Bebbington, Graham Dunn, Daniel Freeman, David Fowler, Elizabeth Kuipers, Philippa Garety

**Affiliations:** a King׳s College London, Institute of Psychiatry, Department of Psychology, London, UK; b School of Medicine, Health Policy and Practice, University of East Anglia, Norwich, UK; c Division of Psychiatry, UCL, London, UK; d Centre for Biostatistics, Institute of Population Health, The University of Manchester and Manchester Academic Health Science Centre, Manchester, UK; e Department of Psychiatry, University of Oxford, Oxford, UK; f School of Psychology, University of Sussex, Sussex, UK

**Keywords:** Schizophrenia, Psychosis, Reasoning, Cognitive therapy, IQ

## Abstract

Understanding how people with delusions arrive at false conclusions is central to the refinement of cognitive behavioural interventions. Making hasty decisions based on limited data (‘jumping to conclusions’, JTC) is one potential causal mechanism, but reasoning errors may also result from other processes. In this study, we investigated the correlates of reasoning errors under differing task conditions in 204 participants with schizophrenia spectrum psychosis who completed three probabilistic reasoning tasks. Psychotic symptoms, affect, and IQ were also evaluated. We found that hasty decision makers were more likely to draw false conclusions, but only 37% of their reasoning errors were consistent with the limited data they had gathered. The remainder directly contradicted all the presented evidence. Reasoning errors showed task-dependent associations with IQ, affect, and psychotic symptoms. We conclude that limited data-gathering contributes to false conclusions but is not the only mechanism involved. Delusions may also be maintained by a tendency to disregard evidence. Low IQ and emotional biases may contribute to reasoning errors in more complex situations. Cognitive strategies to reduce reasoning errors should therefore extend beyond encouragement to gather more data, and incorporate interventions focused directly on these difficulties.

## 1 Introduction

In cognitive models of psychosis, reasoning errors are hypothesised to contribute to both the onset and the maintenance of delusions (e.g. Garety et al., 2007). People with psychosis are held to be prone to reasoning errors because of the increased frequency of characteristic cognitive biases and information processing difficulties associated with psychosis (Hemsley and Garety, 1986; Garety and Freeman, 1999, 2013; Freeman, 2007, So et al., 2010). Psychological interventions designed to help people with delusions therefore target cognitive biases. The aim of the intervention is to modify or compensate for the biases, in order to reduce reasoning errors, and thereby facilitate belief change (e.g. Fowler et al., 1995). However, the effect sizes achieved by these interventions remain small, indicating the need for further development (NICE, 2009). Therapy advances could be informed by progress in two key areas. First, a better understanding of the nature and strength of the relationship between cognitive biases and reasoning errors would inform how much emphasis to place on cognitive biases when intervening with delusions. Second, an investigation of the contribution of other processes to reasoning errors would identify additional foci for intervention.

In this study, we set out to test the association between cognitive biases and reasoning errors, and to investigate the contribution to reasoning errors of other candidate psychological processes. We chose to investigate the ‘jumping to conclusions’ (JTC) data-gathering bias as this is the cognitive bias most robustly associated with psychosis. The bias manifests as the tendency to make hasty decisions with certainty on the basis of little evidence, and is reliably found in approximately half of people with schizophrenia. It is particularly associated with delusions. Around a fifth of the general population also jump to conclusions, and this percentage is increased in the context of high delusional ideation, an at-risk mental state, and in those who have recovered from psychosis (Garety and Freeman, 2013; Garety et al., 2011; Fine et al., 2007; Peters and Garety, 2006; Peters et al., 2008; Woodward et al., 2009; van Dael et al., 2006; Broome et al., 2007; Freeman et al., 2005). The bias is usually assessed by the probabilistic reasoning paradigm, which involves weighing evidence to make decisions, for example, deciding from which of two jars of coloured beads a particular series of beads is being drawn. Most versions of the task employ neutral and abstract material (such as coloured beads) to minimise the influence of other social, affective and cognitive factors on reasoning. The jars may contain beads in an easily discriminable (e.g. 85:15) ratio, or in proportions that are less readily discriminable (e.g. 60:40). The sequence of draws and the ‘correct’ response are usually predetermined, allowing manipulation of the degree of ambiguity of the evidence, and its information value. Emotional versions of the task have been developed, using, for example, words describing personality attributes selected from a survey. The JTC bias has been operationally defined as reaching a decision after fewer than three beads or words (e.g. Garety et al., 2005).

New psychological interventions for people with delusions, designed to specifically target cognitive biases, have been demonstrated to reduce the tendency to JTC on probabilistic reasoning tasks, by extending data-gathering. Such interventions lead to small, but clinically meaningful, changes in delusional beliefs and in quality of life (Woodward et al., 2009; Moritz et al., 2011, 2013; Ross et al., 2011; Waller et al., 2011; Garety et al., 2011). The implication is that reasoning errors, which usually act to maintain delusions, are reduced by extending data-gathering, thereby facilitating belief change.

Accounts of probabilistic reasoning in psychosis (White and Mansell, 2009; Moutoussis et al., 2011) identify three key mechanisms contributing to hasty decision making and linking it to reasoning errors: the information processing difficulties associated with acute psychotic symptoms, leading to reliance on salience at the expense of context; neurocognitive deficits or poor task understanding; and affective or motivational processes, such as threat-related processing or impulsivity. Evidence to date suggests a multifactorial model: the JTC bias is not restricted to those with acute psychosis (Garety et al., 1991; Hemsley, 2005; Peters and Garety, 2006; So et al., 2010; Speechley et al., 2010; Balzan et al., 2012), neurocognitive deficits or limited understanding (Freeman et al., 2008; van Dael et al., 2006; Garety et al., 1991, 2005; Bentall et al., 2009; Lincoln et al., 2010a; Menon et al., 2006; Mortimer et al., 1996; Dudley et al., 2011; Ormrod et al., 2012; Garety et al., 2013). Emotional content increases the tendency to JTC in all participants, irrespective of the presence of psychosis (Dudley and Over, 2003; Fine et al., 2007; Ellett et al., 2008; So et al., 2008; Lincoln et al., 2010b; Freeman et al., 2006; Colbert and Peters, 2002).

However, although task characteristics appear to be of little significance in the tendency to JTC (Garety et al., 2005; So et al., 2012), there is evidence for a psychosis-specific differential effect of task difficulty (ratio discriminability, ambiguity of evidence) and of both emotional and perceptual salience on reasoning errors (e.g. Speechley et al., 2010; Lincoln et al., 2010a; Dudley et al., 2011: Warman et al., 2007; Ormrod et al., 2012). This in turn suggests that reasoning errors may be influenced by psychological factors other than the tendency to make hasty decisions.

A comprehensive analysis of the factors contributing to reasoning errors, both between participants and between different tasks, should help to clarify the relative contributions of different psychological processes, and thereby increase the effectiveness of the new, brief cognitive interventions aimed at improving reasoning skills in psychosis.

### 1.1 The current study

We set out to investigate, in people with psychosis, the association of reasoning errors with the JTC bias (hasty decisions, based on limited data), positive psychotic symptoms (as an index of a salience-based information processing style), emotional processes and IQ on probabilistic reasoning tasks that differed in ratio discriminability, ambiguity of the evidence, and emotional content. The sample partly overlapped with that of So et al. (2012), but the focus of the two studies was entirely different: a detailed analysis of the process correlates of reasoning errors between tasks has not previously been undertaken.

We considered reasoning errors in two ways: firstly, at the level of the participant, identifying the characteristics of those prone to making reasoning errors; and secondly at the level of the individual response on each task (three per participant), identifying differences in the correlates of errors between tasks.

We tested two hypotheses:

(1) the JTC bias will be associated with reasoning errors
(2) independently of their association with the JTC bias, reasoning errors will show task-specific associations with the other psychological processes implicated in probabilistic reasoning in people with psychosis. Specifically, we hypothesised that: (i) reasoning errors on the emotional version of the task would be more influenced by affective processes; (ii) cognitive deficits would play a more significant role on harder (high ambiguity, low discriminability) versions of the task; and (iii) on easy (low ambiguity, high discriminability), neutral versions of the task, in the context of reduced influence of cognitive deficits and emotional factors, reasoning errors would be primarily influenced by levels of positive psychotic symptomatology, as an index of the salience-based information processing style associated with acute psychosis.

## 2 Methods

### 2.1 Participants

The 204 individuals with a recent relapse of psychosis who took part in the current study represented all participants who completed a probabilistic reasoning task in the Psychological Prevention of Relapse in Psychosis Trial (PRP, Garety et al., 2008; ISRCTN83557988). The PRP Trial was a UK-based multicentre randomized controlled trial of cognitive behavioural therapy and family intervention for psychosis, recruiting in four National Health Service Trusts in London and East Anglia. Trial inclusion criteria were: aged between 18 and 65 years; a second or subsequent episode of psychosis starting not more than 3 months before consent to enter the trial; a current diagnosis of non-affective psychosis (schizophrenia, schizoaffective psychosis, delusional disorder), according to ICD-10 criteria and confirmed by SCAN interview conducted by trained raters (Schedules for Clinical Assessment in Neuropsychiatry, WHO, 1992a, 1992b); and a rating at initial assessment of at least 4 (moderate severity) on at least one positive psychotic symptom in the Positive and Negative Syndrome Scale (PANSS, Kay, 1991). The exclusion criteria comprised: a primary diagnosis of alcohol or substance dependency; organic syndrome or learning disability; inadequate command of English to engage in psychological therapy with an English-speaking therapist; unstable residential arrangements. For the current study, all participants scored on a delusion item of the SCAN (Section 19, rating≥1) or PANSS (Items 1, 5 or 6>1) during their trial participation.

### 2.2 Measures

Demographic information (age, self-reported ethnicity, length of illness, gender) was collected from the participant by self-report and corroborated with the clinical record. Length of illness was defined as the time in years since first presenting to services with a schizophrenia spectrum disorder until the point of consent to participate in the current study.

#### 2.2.1 Positive and Negative Syndrome Scale (PANSS, Kay, 1991).

The PANSS comprises 30 items for rating psychotic symptomatology (seven positive; seven negative; and 16 general symptomatology). Each item is rated on a 7-point scale ranging from 1 (*absent*) to 7 (*extreme*). Symptoms are rated over the past 7 days. The mean of the delusions items from the positive scale (Item 1 (Delusions), Item 5 (Grandiosity) and Item 6 (Suspiciousness/Persecution)) was used as an index of current delusion severity, rated on the same scale; a score of 4 or more (moderate severity) on any of these scales was taken to indicate the current presence of a delusion. The 5-factor solution to the PANSS (van der Gaag et al., 2006) was used in this study, providing factor scores for positive symptoms; negative symptoms; disorganisation; excitement and emotion (anxiety/depression).

#### 2.2.2 Probabilistic reasoning tasks

We used three variants of the probabilistic reasoning task, one easy emotionally neutral task with beads in a high discriminability (85:15) ratio and unambiguous evidence; a hard neutral task with beads in a low discriminability (60:40) ratio with more ambiguous evidence, and a hard emotional task, with personality descriptors in a 60:40 ratio and ambiguous evidence. Participants were told that they could see as many items as they wished before making a decision and to decide only when they were certain. Instructions were standardised and presented on a computer screen using the wording of Garety et al. (2005). Draws, and the ‘correct’ decision, were predetermined, as illustrated in Table 1. In the 85:15 task, the first two beads drawn were of a consistent colour and indicative of the overall correct (mostly orange) jar. At no point during the task did the overall pattern of beads indicate the incorrect (mostly black) jar. In the 60:40 beads task, the first bead indicated the overall correct (mostly blue) jar, but was contradicted by the next bead. As the harder task progressed, the pattern of presented beads mostly favoured the correct jar. However, at one decision point the evidence favoured the incorrect jar (at bead three; two red beads and one blue bead presented), and at three further decision points both jars were equally supported by the evidence (beads two, four and six). The emotional task followed an identical pattern of draws to the hard neutral task, with the ‘mostly negative’ survey designated as correct. Reasoning errors therefore, usually involved choosing the ‘mostly positive’ survey, a tendency which might be influenced by mood.

Using the standard coding of errors (choosing the jar designated ‘incorrect’, Garety et al., 2005), half of the guesses made in response to equivocal evidence are coded as correct decisions, as are correct guesses made before any beads are drawn. Conversely, evidence-based decisions made when the evidence supports the ‘incorrect’ jar are counted as errors. However, in the current study reasoning errors were the focus of attention: we wished to avoid arbitrary labelling of a response as an error, and therefore defined reasoning errors as responses that were not supported by the accumulation of presented evidence (i.e. illogical responses). Thus, on the easy task, choosing the mostly black jar at any point was considered to be a reasoning error, coinciding with the standard coding of errors according to experimentally determined jar. On the hard tasks, choosing the mostly red jar or the positive survey at the first, fifth, or seventh and subsequent draws, the mostly blue jar or the negative survey at the third draw, or either after two, four or six draws was coded as a reasoning error. Decisions made before seeing any bead or word, (i.e. at draw 0) were rated as a reasoning error. Thus, our error coding differed from the standard method for a total of 42 decisions (two on 85:15; 19 on 60:40 beads; 21 on 60:40 emotional, see Table 1). In practice, error frequencies were similar using the two methods (85:15 standard, *n*=36 errors; this study, *n*=38 errors; 60:40 standard, *n*=74 errors; this study, *n*=80 errors; emotional, *n*=58 errors, this study, *n*=53 errors). Repeated measures ANOVA showed no effect of method of error coding (x2: new or standard) on error rates by task (x 3: 85:15, 60:40; emotional) or overall (*F* values all <1.7, p values all >0.2).

Errors were additionally coded according to their type: ‘inconsistent’ errors contradicted the accumulated evidence; ‘equivocal’ errors were made in the context of evidence equally supporting each jar choice. Salient errors were consistent with the current draw, but not indicated by the accumulated evidence and could be either inconsistent (e.g. choosing the black jar on draw 4 (black) in the 85:15 task, after already seeing three orange beads), or equivocal (e.g. choosing the red jar on draw 2 (red) of the 60:40 task, having already seen a blue bead). On the emotional task, errors were also coded by whether or not the positive survey was chosen. Making a decision after fewer than three draws was considered to be a hasty decision, indicative of the JTC bias (Garety et al., 2005; So et al., 2012).

Responses on each task (three responses per participant) were recorded. Participants were grouped dichotomously according to whether they made a reasoning error on any completed task, and according to whether they made a hasty decision (after fewer than three draws) on any completed task.

#### 2.2.3 The Quick Test (Ammons and Ammons, 1962)

The Quick Test was used to provide an estimate of current intellectual functioning (IQ). The task was completed only by those whose first language was English, as it is not standardised for respondents answering in a second language.

### 2.3 Procedure

All measures were completed during a baseline assessment; symptom measures always preceded the reasoning task and the IQ measure. Reasoning tasks were always completed in the same order, beginning with the 85:15 task, then the neutral 60:40 task, then the emotional 60:40 task. Full ethical approval was obtained prior to the onset of the study, and all participants gave informed consent (South East National Research Ethics Service Committee ref. 01/1/14).

### 2.4 Analysis

Analyses were completed using SPSS version 20 (IBM, 2011) and STATA 12 (Statacorp, 2011). All participants completed the 85:15 task, 10 participants did not complete one or both of the other two tasks (*n*=4 60:40; *n*=9 emotional). Participants with any missing data were excluded from relevant analyses, and *n*׳s reported. The clinical and demographic characteristics of participants completing a probabilistic reasoning task (and therefore included in the current study) were compared to those who did not complete a probabilistic reasoning task, using independent sample t-tests and Chi-square tests. For the participant level analyses of errors, two groups were formed according to the dichotomous rating of whether or not the participant made any reasoning errors (1=made a reasoning error on at least one task; 0=no reasoning errors on any completed task). For the response level analyses, decisions were coded dichotomously according to whether or not they were an error (1=error; 0=not an error).

For the main analyses, we employed two series of logistic regression analyses to investigate our hypotheses. For the first set of analyses, at participant level, the tendency to make reasoning errors was the dependent variable, and a dichotomised rating of JTC was the predictor variable (1=JTC on at least one task; 0=no JTC on any task). Demographic and clinical variables (age, self-reported ethnicity (coded as White/Other), length of illness, gender), PANSS factor scores, IQ, and delusion status were controlled.

The second hypothesis concerned differences in responses between tasks and therefore required three separate repeated measures analyses, each focusing on one of the hypothesised predictors of reasoning errors: IQ, PANSS Positive or PANSS Emotion. The dependent variable in all analyses was whether or not a reasoning error was made. Task type formed a within subjects variable with three levels, taking the easy neutral task (85:15 beads) as the reference category, and comparing firstly to the 60:40 task (Task 2) then the emotional task (Task 3). The independent variable of primary interest in each analysis was the interaction between the relevant predictor and task type, tested using an interaction term (predictor x task type), controlling for predictor, task type, JTC, and demographic variables. We checked for associations between JTC and predictor variables; none were found (ORs all equal to 1.0, *p* values>0.3). Analyses were also repeated excluding JTC and demographic variables, with identical results, therefore only results from the planned, fully controlled analyses are reported. Post-hoc correlational analyses examined the variation in reasoning errors with speed of decision making, and in delusion severity according to error type, controlling for the tendency to make hasty decisions.

## 3 Results

### 3.1 Demographic and clinical characteristics

These are shown for the 204 participants who completed a probabilistic reasoning task (and therefore participated in the current study) in Table 2. The 97 participants in the PRP trial who did not complete a probabilistic reasoning task and therefore were not included in the current study did not differ on any demographic variable, but did have a lower IQ score (Mean=88.9, S.D.=16.3, *n*=43, *t*=−2.3, d.f.=234, *p*=0.02). Clinically, they scored higher on Excitement (Mean=5.8, S.D.=2.6, *n*=97, *t*=2.3, d.f.=145, *p*=0.02) and Disorganisation (Mean=18.0, S.D.=6.1, *n*=97, *t*=−2.0, d.f.=299, *p*=0.04). No significant differences were found for age, gender, length of illness, ethnicity, diagnosis, delusion severity, or any other PANSS factor score (*t* values<1.5, *χ*^2^ values<6.0, *p* values<0.05). Mean delusion severity was 3.3 (S.D.=1.2); the majority of participants (*n*=163) had a current delusion of at least moderate severity on the PANSS.

### 3.2 Pattern and correlates of reasoning errors at participant level across all tasks

Of all participants, half made at least one reasoning error (*n*=106/204, 52%). Of these, 76 (72%) also jumped to conclusions. Of those not making any reasoning errors (*n*=98), 45 (46%) jumped to conclusions. Table 2 shows the demographic and clinical characteristics of those making and not making reasoning errors. Binary logistic regression showed that the tendency to JTC was a significant predictor of the tendency to make reasoning errors, with those showing the JTC bias being more than three times as likely to make an error as those not jumping to conclusions (OR=3.2, 95% CI 1.6 to 6.1, *p*=0.001), irrespective of controlling for PANSS factor scores, demographic variables and IQ. Those making reasoning errors did not differ from those not making reasoning errors on any other demographic or clinical variable, irrespective of controlling for the tendency to JTC (ORs range from 0.6 to 1.0; *t*-values<1.8; *χ*^2^ values<1.6; *p* values<0.05). The pattern was the same irrespective of controlling for current delusion status. Those with a current delusion were almost four times as likely to make an error than those without (OR=3.8, 95% CI=1.2 to 11.9, *p*<0.05) (Table 3).

### 3.3 Pattern and correlates of reasoning errors at response/task level

Table 1 shows the frequencies of each response by draw and task. Just over a quarter of all responses (171/599) were reasoning errors, and almost half of all responses were hasty (255/599), irrespective of whether or not they were correct. Over half of all reasoning errors (96/171, 56%) were inconsistent (made in direct contradiction to the evidence presented; e.g. choosing the mostly black jar, having seen only orange beads). The remainder were made in the context of no data (13/171, 8%), or equivocal data (62/171, 36%). Just over a fifth of reasoning errors (38/171, 22%) were attributable to salience, almost all in the context of equivocal data (*n*=35).

We examined the variation in the association of reasoning errors with each potential psychological mechanism between tasks, using repeated measures binary logistic regression, with the 85:15 task as the reference category, compared firstly to the 60:40, then to the emotional task. As hypothesised, there were significant interactions between task type and IQ, PANSS Positive and PANSS Emotion, independently of controlling for JTC and demographic variables (Table 4). JTC remained a significant predictor of reasoning errors in each repeated measures analysis (ORs ranged from 2.1 to 2.4, *p* values≤0.001). Differences were predominantly between the easy and hard neutral tasks. Reasoning errors were more common on the hard neutral task, irrespective of whether they were hasty or not, but not on the emotional task, which did not differ from the easy neutral task. Mean scores showed that reasoning errors on the easy neutral task (which were all inconsistent) were associated with higher levels of PANSS Positive than on the hard neutral task, while reasoning errors on the latter were associated with lower IQ and lower PANSS Emotion scores (Table 5). Frequency and severity of delusions were highest for the hasty errors group on the 85:15 task, and the hasty correct group (those choosing the negative survey) on the emotional task.

Post-hoc correlations, controlling for the tendency to JTC, were used to identify the pattern of associations with error type. On the 85:15 task, hasty inconsistent errors were associated with higher IQ (*r*=0.2, *p*=0.005, *n*=187), positive symptoms (*r*=0.2, *p*=0.003, *n*=201) and delusion severity (*r*=0.2, *p*=0.01, *n*=201). On the 60:40 task, hasty inconsistent errors were associated with low levels of IQ (*r*=−0.2, *p*=0.03, *n*=184) and negative affect (*r*=0.2, *p*=0.02, *n*=197), but not delusion severity. On the emotional task, both equivocal and salient positive errors were associated with positive symptoms and delusion severity (r values all −0.2, *p* values≤0.02, *n*=192), irrespective of whether they were hasty or not; salient positive errors were also associated with lower levels of negative affect (*r*=−0.14, *p*<0.05, *n*=192). No other error type showed an association with any of the psychological mechanism variables, or with delusion severity (*r* values<0.15, *p* values>0.05). The rate of reasoning errors clearly decreased as the number of draws to decision increased, showing small but consistent bivariate correlations overall (*r*=−0.22, *p*<0.001, *n*=599), and for each task (*r* values range from 0.2 to 0.3, *p* values<0.05).

## 4 Discussion

We set out to investigate the pattern and correlates of reasoning errors under differing task conditions, with the aim of testing the association between the jumping to conclusions (JTC) bias (both the tendency to make hasty decisions and for those decisions to be based on limited data) and reasoning errors, and of identifying other processes contributing to reasoning errors. The purpose was to inform the further development of targeted cognitive therapy interventions designed to facilitate helpful belief change for people with delusions.

We found strong associations between the tendency to make reasoning errors and to make hasty decisions: the rate of reasoning errors clearly decreased as the number of draws considered increased. These findings support current models of intervention, and suggest that helping people to gather more information should reduce reasoning errors, and consequently facilitate changes in delusions.

However, reasoning errors were associated with current delusions, irrespective of making hasty decisions and half of the reasoning errors were not hasty, suggesting additional routes by which reasoning errors may arise, and influence delusions. Two thirds of hasty errors, and around half of the total reasoning errors were inconsistent, directly contradicting all the available evidence (for example, seeing one or more orange beads and then deciding on the black jar). Although this tendency has been noted previously (Lincoln et al., 2010a), the error rate was much higher in our group. This was not the result of our particular methods of coding (rates were slightly higher using standard coding, see Table 1), but may reflect the clinical presentation of our participants, who had a history of delusions, a recent experience of relapse, and continuing positive symptoms.

The tendency to make inconsistent errors was most evident on the easy neutral task, on which some participants made contradictory decisions despite successive pieces of evidence consistently indicating the other jar. Participants showing this tendency had high levels of delusions, consistent with a maintenance role. They also had high IQ scores, suggesting the behaviour did not result from lack of ability or understanding. Although the tendency was associated with positive symptoms, contrary to hypothesis, salience did not appear to be the mechanism underlying these errors. Rather, our findings suggest other positive symptom related processes, such as suspiciousness of presented evidence, or overconfidence in guesses or ‘gut feelings’ (Andreou et al., 2014; Köther et al., 2012; Freeman et al., 2012; Moritz and Woodward, 2006).

On the hard neutral task, in contrast, lower IQ was associated with reasoning errors, suggesting that poor task understanding, or difficulties with the nature of evidence-based reasoning may exert more influence when the task is harder (less discriminable ratio; more ambiguous evidence). These errors were primarily inconsistent, and were also associated with lower levels of negative affect, but not with delusions.

Error rates were lower on the emotional task, despite the same levels of difficulty as the hard neutral task, raising the possibility that negative affective biases may reduce the likelihood of choosing the positive survey, irrespective of the evidence. Consistent with this, those making hasty but correct decisions on the emotional task showed higher levels of delusion severity; while choosing the positive survey, when presented with 50:50 evidence or a positive word, was associated with lower levels of delusions, and, for salient errors, with less negative affect.

The tendency to make reasoning errors was not associated with the PANSS Excitement factor score, arguing against a role for impulsivity. Salience-based reasoning appeared to account for around a fifth of errors, across all tasks, rather than just on the easy neutral task, but other than the content specific association with delusions, was not associated with any of the predictor variables, or with delusion severity.

### 4.1 Clinical implications

The findings have implications for cognitive behavioural interventions for people with delusions. Around half of the reasoning errors resulted from a tendency to make a decision which directly contradicted the presented material, even in the face of repeated evidence to the contrary. This seemed to be particularly characteristic of people with positive symptoms on the easy task. It was not associated with lower IQ. The findings raise the possibility that this tendency sometimes contributes to delusion maintenance. Simply accruing more evidence may not help these people, and an alternative strategy to data-gathering may be needed. Working directly on beliefs about the nature of evidence and reasoning, and the meaning of a particular piece of evidence might be useful. A different subset of participants made reasoning errors on the hard task, and had lower IQ scores. General remedial strategies, in addition to addressing data-gathering, may be useful for these people, who may be making reasoning errors simply due to lack of understanding, or poor concentration. This tendency was not associated with delusions. Affective biases may have acted to reduce errors on the emotional task in this study, and could also therefore influence delusional reasoning.

### 4.2 Limitations

The study has a number of limitations. Participants were selected not only for a research study, but also for ability to complete a particular task, and may not, therefore, be representative of all people with similar diagnoses. The measure of IQ is limited, and replication with a more comprehensive assessment of neuropsychological functioning would allow stronger conclusions to be drawn. We carried out multiple comparisons, and while the association of JTC with reasoning errors is well-established, the other findings require replication. Task understanding was not formally assessed, and although apparently ‘illogical’ decisions were associated with high IQ, it remains possible that participants had simply misunderstood the nature of the task. However, poor task understanding is not always readily distinguishable from a ‘psychotic-like’ performance, which, by definition implies a limited influence of current evidence on decision-making. We employed a novel coding of errors, and, while we considered this coding to have more validity (in that it was directly linked to what each participant had seen, rather than experimentally determined), results were in fact almost identical to those that would have been found using standard coding of errors, and suggests that recoding may not be necessary. For the emotional task in particular, standard coding preserves the link with content and may therefore be superior. Associations were cross-sectional, so may not be causal, and the term ‘predictor’ is used in a statistical sense only. A longitudinal study of changes in delusions in relation to reasoning errors would help in testing causal relationships. Our cross-sectional design did not permit experimental manipulation of task parameters, such as ratio discriminability and ambiguity of the evidence presented, which could also clarify causal relationships. Nevertheless, the tendency to make contradictory decisions is clear, and has not previously been investigated in the literature.

### 4.3 Conclusions

Reasoning errors are associated with hasty decision making, but only the minority of reasoning errors result from being misled by limited data. A high proportion of reasoning errors directly contradicted the evidence presented, particularly on the easy task, where this tendency was associated with positive symptoms and higher functioning. In contrast, reasoning errors on the hard task were associated with low IQ. Direct interventions focused on the meaning and use of evidence, and not just on gathering more data, may be a helpful addition to cognitive behavioural reasoning interventions.

## Conflict of interest

None.

## Figures and Tables

**Table 1:** Pattern of draws in each probabilistic reasoning task and frequency counts of decisions by draw at response level (*n*=599 responses across *n*=204 participants).



*Key: Grey shading=‘Error’; JTC: Jumping to Conclusions data-gathering bias; ^a^Decisions made without seeing any data*.

**Table 2:** Demographic and clinical characteristics according to the presence of reasoning errors at participant level.

	**Total**	**Reasoning error**	
		**No**	**Yes**
	*n*=204	*n*=98	*n*=106
	Mean (S.D.)/*n* (%)		
JTC (%JTC/no JTC)	59/41	46/54	72/28
IQ (Quick Test)^a^	94.4	96.2	92.7
	(14.0)	(14.1)	(13.8)
Age (years)	37.5	36.8	38.2
	(11.1)	(10.9)	(11.3)
Gender (% male/female)	72/28	73/27	70/30
Ethnicity (%BME/non-BME)	27/73	24/76	31/69
Length of illness (years)^b^	10.4	10.1	10.6
	(8.4)	(8.5)	(8.4)

**PANSS factors**			
Emotion	10.3	10.6	10.0
	(4.3)	(4.6)	(4.0)
Negative	13.4	13.7	13.1
	(6.6)	(6.4)	(6.8)
Excitement	5.1	5.1	5.1
	(1.9)	(1.8)	(1.9)
Disorganisation	16.7	16.0	17.3
	(5.2)	(5.1)	(5.2)
Positive	18.6	18.3	18.9
	(5.7)	(5.8)	(5.6)
Delusions	9.8	9.8	9.8
	(3.6)	(3.6)	(3.6)
Other	8.1	7.7	8.4
	(2.7)	(2.8)	(2.7)
% Current delusion	80	82	78

**Diagnosis** (%)			
ICD F20	83	82	84
ICD F22	1	1	1
ICD F25	16	17	15

a Error *n*=97; no error *n*=93.

b Error *n*=104; no error *n*=9; S.D.=standard deviation; JTC=Jumping to conclusions data gathering bias; IQ=Intelligence quotient; BME=Black and minority ethnic; PANSS=Positive and Negative Syndrome Scale (Kay, 1991); ICD=International Classification of Disease (WHO, 1992a, 1992b).

**Table 3:** Frequency of errors by type for each probabilistic reasoning task at response level (*n*=599 responses across *n*=204 participants).

	**All (599)**	**8515 (204)**	**6040 (200)**	**Emot (195)**	**Emot +ve (195)**
**Total errors**	171	38	80	53	32
% of total responses	28%	19%	40%	27%	16%
Hasty responses (<3 draws)	255	105	78	72	
% of total responses	42%	51%	39%	37%	
**Hasty errors**	92	27	41	24	19
% of total errors	54%	71%	51%	45%	36%
% of total hasty responses	36%	26%	53%	33%	26%
No data	13	4	6	3	2
% hasty errors	14%	15%	15%	12.5%	8%
Inconsistent with data	58	23	23	12	12
% hasty errors	63%	85%	56%	50%	50%
Equivocal data	21	0	12	9	5
% hasty errors	23%		29%	37.5%	9%
Salient (all equivocal)	13	0	8	5	5
% hasty errors	14%		19%	21%	21%

**Non-hasty errors**	79	11	39	29	13
% of total errors	46%	29%	49%	54%	24%
% of total non-hasty responses	23%	11%	32%	24%	11%
Inconsistent with data	38	11	16	11	4
% non-hasty errors	48%	100%	41%	38%	31%
Equivocal data	41	0	23	18	9
% non-hasty errors	52%		59%	62%	69%

Salient	25	4	11	10	4
% non-hasty errors	32%	36%	28%	35%	31%
Inconsistent with data (%)	5 (20%)	4 (100%)	0	1 (10%)	1 (25%)
Equivocal data (%)	20 (80%)	0	11 (100%)	9 (90%)	3 (75%)

*Key*: *Emot*: *Emotional task*.

**Table 4:** Binary logistic regression at task level showing differences between tasks in the association of clinical variables with reasoning errors (*n*=599 decisions across *n*=204 participants).

**Independent variables**^a^	**Predictor**^a^		
	**IQ**	**PANSS Positive**	**PANSS Emotion**
	**OR (95% CI)**	**OR (95% CI)**	**OR (95% CI)**
	*p*	*p*	*p*
**JTC bias**	2.4	2.1	2.2
	(1.5–3.8)	(1.4–3.3)	(1.4–3.4)
	<*0.001*	*0.001*	<*0.001*

**Predictor**	1.0	1.1	1.0
	(1.0–1.1)	(1.0–1.1)	(0.9–1.1)
	*0.2*	*0.05*	*0.5*

**Predictor×Task 2 (60:40)**^**2**^	0.9	0.9	0.9
	(0.92–0.98)	(0.86–0.99)	(0.82–0.97)
	*0.003*	*0.03*	*0.01*

**Predictor×Task 3 (emot)**^b^	1.0	0.9	1.0
	(0.9–1.0)	(0.9–1.0)	0.9–1.1
	*0.5*	*0.13*	*0.8*

*Key*: *PANSS*: Positive & Negative Syndrome Scale (Kay, 1991); JTC: Jumping to Conclusions data-gathering bias.

a Controlling for Age, Gender, Ethnicity, Length of Illness, Task.

b Reference category, 85:15 task; Emot: Emotional task; for relevant means see Table 4.

**Table 5:** Mean differences between tasks in the association of clinical variables with the tendencies to jump to conclusions and to make reasoning errors.

**Task**		**Mean (S.D.)**						
		***n***	**IQ**	***n***	**PANSS emotion**	**PANSS positive**	**Current delusion %**^a^	**Delusion severity**
**Error**								
**JTC**	**85:15**	26	99.4 (13.4)	27	10.0 (4.2)	21.3 (5.6)	89	11.3 (3.6)
	**60:40**	39	90.7 (14.5)	41	9.2 (3.6)	19.0 (5.7)	78	10.0 (3.9)
	**Emot**	22	95.1 (13.9)	24	10.3 (4.0)	19.4 (5.4)	79	9.8 (3.7)

**No JTC**	**85:15**	10	90.6 (17.3)	11	12.3 (4.2)	17.8 (3.3)	82	9.1 (2.7)
	**60:40**	34	91.1 (13.2)	39	9.6 (4.0)	17.8 (5.2)	74	9.3 (3.3)
	**Emot**	26	95.1 (11.2)	29	10.3 (4.7)	18.1 (6.3)	72	9.4 (3.8)

**No error**								
**JTC**	**85:15**	73	91.0 (12.9)	78	10.4 (4.4)	18.6 (5.5)	81	9.8 (3.7)
	**60:40**	34	95.6 (13.0)	37	11.4 (4.2)	19.3 (5.7)	84	9.9 (3.7)
	**Emot**	44	92.2 (15.1)	48	9.8 (4.2)	19.6 (5.2)	88	10.3 (3.4)

**No JTC**	**85:15**	81	96.3 (14.1)	88	10.1 (4.3)	17.9 (6.0)	76	9.4 (3.7)
	**60:40**	80	97.2 (14.1)	83	10.6 (4.7)	18.5 (6.0)	82	10.0 (3.7)
	**Emot**	91	95.3 (14.2)	94	10.4 (4.3)	18.1 (6.0)	77	9.7 (3.8)

*Key*: *IQ*: *Intelligence Quotient as measured by the Quick Test*; *PANSS*: Positive & Negative Syndrome Scale (Kay, 1991).

a Current delusion of moderate severity >3 on any PANSS delusion item (1, 5 or 6); Emot: Emotional Task; JTC: Jumping to Conclusions data-gathering bias.
